# Effects of Indwelling Pleural Catheter on Severe Acute Pancreatitis: A Retrospective Study

**DOI:** 10.1155/2022/1919729

**Published:** 2022-01-27

**Authors:** Qian Yao, Siyang Peng, Yanping Wu, Pi Liu

**Affiliations:** Department of Gastroenterology, The First Affiliated Hospital of Nanchang University, Nanchang, Jiangxi, China

## Abstract

**Background:**

Pleural effusion (PE) is an important predictor for severity and prognosis of severe acute pancreatitis (SAP). However, there are few studies focused on the effects and timing of indwelling pleural catheter (IPC) on SAP. Considering this, we designed a retrospective study to verify the relationship between PE and severity of SAP and observe the effects and timing of IPC in SAP.

**Methods:**

A total of 309 SAP patients were enrolled. Based on the presence or absence of PE and IPC and IPC time, the enrolled patients were divided into 6 groups. Then, baseline parameters, disease severity, critical complications, ventilator supporting time (VST), length of stay (LOS), and 60-day mortality were compared between each two groups.

**Results:**

PE was a risk factor for death of SAP, but not an independent risk factor. SAP patients with PE rather without PE had higher critical complication rates (*p* < 0.001), along with longer VST (*p* < 0.001) and LOS (*p* < 0.001). And the critical complication rates were lower in group 1 (IPC within 1 week of onset) than group 2 (IPC after 1 week of onset). Further, patients in group 1 also had shorter LOS (*p* = 0.042) and VST (*p* = 0.001) than those in group 2. In addition, the survival analysis showed the risk of death in the PE group was higher than the non-PE group (HR 6.6, 95% CI, 3.67–11.86, and *p* < 0.001). And the risk of death in group 1 was lower than group 2 (HR 0.26, 95% CI, 0.08–0.84, and *p* = 0.025).

**Conclusions:**

PE is a risk factor for death of SAP, but not an independent risk factor. IPC, especially IPC within 1 week of onset, has clinical practical value in SAP.

## 1. Introduction

Acute pancreatitis (AP) is an acute inflammatory process involving the pancreas and extrapancreatic tissue, and its global incidence is increasing [1]. About 20% of AP patients develop severe acute pancreatitis (SAP), with mortality approaching 40% [2]. A prominent feature of early SAP is distant organ dysfunction, particularly the lung [3, 4]. The intrathoracic complications of SAP include the pleura, lung, and heart [5]. With the continuous aggravation of intrathoracic complications, they can be further developed into acute respiratory distress syndrome (ARDS), which is life-threatening [6]. Studies have shown 60% of SAP patients died within one week of onset, while the incidence of pleuropulmonary complication was up to 94% [5, 7].

Pleural effusion (PE) is a common intrathoracic complication in SAP [5]. Recent report suggests that the incidence of PE in AP is up to 50% [8]. Besides, PE is a good predictor for SAP and even better than Ranson score and Acute Physiology and Chronic Health Evaluation II (APCHE II) score in reflecting the severity of SAP [8, 9]. Indwelling pleural catheter (IPC) is effective for benign and malignant PE and could improve living quality of patients by intermittently relieving PE symptoms [10, 11]. So far, PE is studied primarily as a predictor in SAP, but the effects and timing of IPC in SAP remain unclear. To address this issue, we conducted this study to verify the relationship between PE and the severity of SAP and focus on the effects and timing of IPC in SAP.

## 2. Materials and Methods

### 2.1. Patients

The Ethics Committee of the First Affiliated Hospital of Nanchang University had approved the protocol of this study. Because of the retrospective nature of the study, we gave written informed consent. Between January 1, 2017, and January 1, 2021, AP patients were evaluated within 72 hours after admission according to the inclusion and exclusion criteria (Table 1).

### 2.2. Definition

Patients with two or more of the following three manifestations were diagnosed with AP: (1) imaging (ultrasound or computed tomography) indicates AP; (2) acute epigastric pain; (3) serum enzyme level was more than 3 times higher than normal [12]. And PE was diagnosed by imaging examination (X-ray, CT, and ultrasound) on admission.

### 2.3. Severity Assessment

Based on the revised Atlanta classification, AP was classified as mild AP, moderate AP, and SAP [12]. The modified Marshall score ≥ 2 is considered to be organ failure, and it is recommended to assess respiratory, circulatory, and renal failure in AP [12].

The Extra Pancreatic Inflammation on CT (EPIC) score is based on the presence of signs of extrapancreatic inflammation, which is range from 0 to 7. For predicting the severity and prognosis of SAP, EPIC score ≥ 4 has a 100% sensitivity and is better than the Balthazar score and CT Severity Index [13]. Therefore, EPIC score ≥ 4 was included as inclusion criteria to reflect the severity of patients included in this study.

### 2.4. Biochemical Indicators

The laboratory indicators (WBC, C-reactive protein, procalcitonin, D-dimer, lactate dehydrogenase, and creatinine) were obtained from blood samples taken from enrolled patients within 48 hours of symptom onset.

### 2.5. Intervention Indication

The following two conditions need to be met during IPC: (1) imaging showed the fluid thickness on thoracic ultrasound > 1 cm; (2) there was a safe puncture path. IPC process was described in more detail in earlier study [14]. The signs of IPC success are as follows: (1) the drainage fluid could be seen by redrawing after successful puncture; (2) the catheterization could be seen in the target area under imaging.

### 2.6. Grouping

The 309 SAP patients selected were divided into the PE and non-PE groups. Then, patients in the PE group were divided into the IPC and non-IPC groups, and IPC group patients were divided into the IPC within 1 week of onset and IPC after 1 week of onset groups.

### 2.7. Follow-Up

The follow-up data for this study were the patients' 60-day mortality. The 60-day mortality data could be collected from the hospitalized cases, telephone, or outpatient (see supplementary material (available here) for follow-up details).

### 2.8. Primary and Secondary Endpoints

Primary endpoints are as follows: the 60-day mortality and LOS. Secondary endpoints are as follows: (1) abdominal compartment syndrome (ACS), (2) acute necrosis pancreatitis (ANP), (3) organ failure, (4) fungus infection, (5) bleeding and thrombus, (6) VST, and (7) percutaneous catheter drainage (PCD) and minimally invasive debridement (MID).

#### 2.8.1. Statistical Analysis

Quantitative variables were analysed by ANOVA, and Fisher exact test was used to compare the categorical variables. The factors related to death of SAP were determined by Cox proportional hazards models. Due to our study had an exploratory nature, relevant clinical or biochemical variables (Table 2) were evaluated as potential confounding factors. And the risk of death was estimated by Kaplan-Meier; the differences between each two groups were evaluated by log-rank test. *p* < 0.05 stood for statistically significant. All statistical analyses were performed by SPSS 26.0 (IBM, Armonk, NY, USA), and the survival curve graphs were drawn by GraphPad Prism 8 (GraphPad Software Inc., San Diego, CA, USA).

## 3. Results

### 3.1. Selected Patients

**Figure 1:**
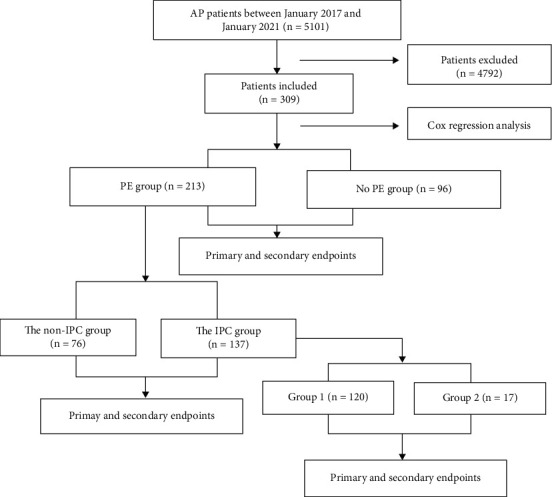
Flowchart of study. PE: pleural effusion; IPC: indwelling pleural catheter. Group 1: IPC within 1 week of onset. Group 2: IPC after1 week of onset.

Figure 1 shows the flow-chart of study. Between January 1, 2017, and January 1, 2021, there were 5101 AP patients in our digestive centre. After screening, a total of 309 SAP patients were enrolled for meeting the inclusion and exclusion criteria (Table 1 and Figure 1).

### 3.2. Cox Regression Analysis

PE was a risk factor for death of SAP, but it was not an independent risk factor. In addition, APACHE II score, EPIC score, modified Marshall score, CRP, WBC, Ca, Lac, amylase, fungal infection, bleeding, ACS, and organ failure were also the risk factors of death for SAP. However, only modified Marshall score, ACS, bleeding, and organ failure were the independent risk factors (Table 2).

### 3.3. Baseline Characteristics

Patients in the PE group were more serious than those in the non-PE group. APACHE II score (11.9 ± 4.3 vs. 14.1 ± 7.4, *p* = 0.02) and serum creatinine values (157.6 ± 141.8 vs. 208.4 ± 177.1, *p* = 0.02) were lower in the IPC group than the non-IPC group. All the baseline variables were balanced between group 1 and group 2 (Table 3).

### 3.4. Primary Endpoints

**Figure 2:**
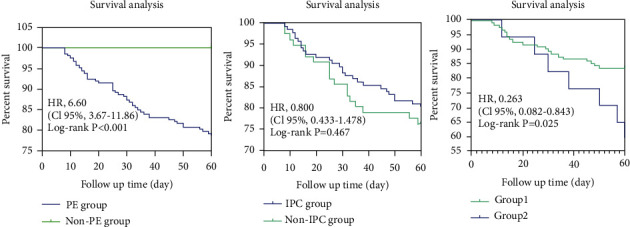
Kaplan-Meier percent survival curves of 60 days. PE: pleural effusion; IPC: indwelling pleural catheter. Group 1: IPC within 1 week after onset. Group 2: IPC after 1 week of onset.

The risk of death in the PE group was higher than the non-PE group (HR 6.6, 95% CI, 3.67–11.86, and *p* < 0.001). And the risk of death of group 1 was lower than group 2 (HR 0.263, 95% CI, 0.082–0.843, and *p* = 0.025). However, there was no significant difference in risk of death between the IPC and non-IPC groups (Figure 2).

LOS was significantly longer in the PE group than the non-PE group (38.8 days vs. 20.3 days, *p* < 0.001). Further, group 1 versus group 2 had a shorter LOS (37.1 days vs. 51.6 days, *p* = 0.04). However, there was no difference in LOS between the IPC and non-IPC groups (Table 4).

### 3.5. Secondary Endpoints

The rates of multiple organ failure (51.6% vs. 19.8%), bleeding (19.2% vs. 2.1%, *p* < 0.001), fungus infection (20.2% vs. 1%, *p* < 0.001), and necrosis (80.3% vs. 45.8%, *p* < 0.001) were higher in the PE group than the non-PE group. Furthermore, patients with PE rather without PE had longer VST (9.5 vs. 2.0, *p* < 0.001). And incidence of multiple organ failure (54.3% vs. 63.2%, *p* = 0.01) and PCD (47.4% vs. 61.8%, *p* = 0.04) was significantly lower in the IPC group than the non-IPC group. Compared with group 2, group 1 had lower rates of PCD (41.7% vs. 88.2%, *p* < 0.001), MID (19.2% vs. 52.9%, *p* = 0.005), multiple organ failure (41.7% vs. 70.6%, *p* = 0.03), and necrosis (76.5% vs. 94.1%, *p* = 0.03), along with a shorter VST (7.6 vs. 18.9, *p* = 0.001).

## 4. Discussion

SAP is a common gastrointestinal cause of urgent hospital treatment, which has become a difficult problem to be solved clinically [15]. The severity of pancreatitis is not mainly due to the lesions affecting the pancreas, but to the involvement of the extrapancreatic organs, especially the lung [3–5]. Intrathoracic complication is a core indicator of SAP severity and is associated with nearly 30% of pancreatitis deaths [5, 16]. PE is one of intrathoracic complications in SAP [17, 18]. The mechanisms of PE in AP include the following: (1) changes in capillary permeability caused by inflammation [19], (2) blockage of the pancreatic duct due to pancreatic duct injury [17, 20], and (3) a sinus was formed between the pancreatic pseudocyst and pleural cavity [17]. In addition, PE is an early indicator for the SAP severity, especially within 24 hours of admission [21, 22]. If PE is small and asymptomatic, it could be managed conservatively. But when there is a tappable fluid (>1 cm thickness), thoracentesis may be considered to rule out an infected PE and release the symptoms of shortness of breath [18]. Since an infected PE was undrained, fibrothorax would be developed [18]. However, there are few studies on the effects and timing of IPC in SAP. The reason is understandable: (1) PE seems to be negligible compared to the other serious complications; (2) since the treatment of PE is relatively simple, researchers have much less interest in treatment of PE than other complications. To provide more clinical evidence on the effects and timing of IPC in SAP, we designed this study to verify the relationship between PE and severity of SAP and focus on the effects and timing of IPC in SAP.

### 4.1. Main Findings

The findings of the study are as follows: (1) PE is a risk factor for death of SAP, but not an independent risk factor; (2) SAP patients with PE rather without PE were more serious and had a worse outcome; (3) compared with IPC after 1 week, IPC within 1 week could reduce LOS, VST, complication rates, and risk of death in SAP.

### 4.2. Comparison with Other Studies

This study showed that SAP patients with PE rather without PE were more serious and had worse outcome, which was consistent with the previous studies [8, 9, 23]. In addition, this study also drew the same results as before: PE was a risk factor for death, but not an independent risk factor for death of SAP [24]. Traditionally, the pancreas necrosis itself was considered an independent determinant of mortality [25]. However, evidence now suggests that necrotizing pancreatitis without organ failure has similar outcome as interstitial pancreatitis, which has a 1-2% mortality [26]. The revised Atlanta guidelines classified the severity of AP as mild, moderate, or severe based on the presence or absence of organ failure, effusions, and comorbidities, rather than on the severity of pancreas [12]. Furthermore, studies reported that complications were predictors of poor prognosis and disease severity for AP [25, 27]. In summary, the severity and mortality of SAP are related not only to the pancreas, but also to the extrapancreatic involved organs [5]. Thus, our results may be credible.

In the study, IPC could reduce the rates of multiple organ failure and PCD, suggesting IPC may have certain clinical value in SAP. The mechanism could be the following: (1) the PE of pancreatitis is usually exudative. When there is a tappable fluid, IPC may be considered to rule out an infected PE [18]; otherwise, the infected PE may develop into a fibrous thoracic cavity, resulting in respiratory disorder [18, 28]; (2) the incidence of PE depends on the severity of inflammation in SAP [18, 19]. There are a lot of inflammatory factors in pancreatic effusions. The risk of death in SAP depends by the entity of “cytokines storm” in the blood and extrapancreatic effusions [29]. In this case, IPC may be reasonable.

There are two mortality peaks in AP, and the first peak is due to multiorgan dysfunction during the first week. Since 60% of AP deaths occurred in the first week [7, 28], we divided the IPC group into the IPC within 1 week of onset and after 1 week of onset groups. Then, we were surprised to find that compared with IPC after 1 week of onset group, IPC within 1 week of onset group had lower 60-day mortality and critical complication rates, along with shorter LOS and VST. The mechanisms may be that SAP is an inflammatory disease, releasing a mass of inflammatory mediators and activating inflammatory cells and cytokines [30]. The risk of death in SAP depends by the entity of “cytokines storm” in the blood and extrapancreatic effusions [29]. Thus, IPC within 1 week could remove cytokines and inflammatory cells from PE in the early stage of pancreatitis, thereby improving the condition. However, the mechanisms need to be further explored.

#### 4.2.1. Limitations

Limitations of the study are as follows: (1) this is a retrospective study, and randomized controlled trials are ideal, but retrospective studies are acceptable when a sufficient number of randomized controlled trials have not been conducted. (2) The study just involves a single digestive centre of Chinese population. (3) The number of patients who received IPC was more than that of non-IPC patients, and the number of patients who received IPC was more than that of IPC after 1 week of onset, and we can explain the difference through the attitudes of doctors and patients in our centre for PE of SAP. When conditions permit, prospective studies could be conducted to strictly control the timing of IPC to increase the reliability of experimental results. (4) Due to the limited time in this study, the sample size collected is small, so more cases can be included in the future study for a deeper and wider research.

Despite these limitations, it still gives clinicians some implications: IPC, especially IPC within 1 week of onset, seems to have clinical practical value in SAP.

## 5. Conclusions

In conclusions, (1) PE is a risk factor for SAP mortality, but not an independent risk factor. (2) IPC, especially IPC within 1 week of onset, seems to have clinical practical value in SAP.

## Figures and Tables

**Table 1:** The inclusion and exclusion criteria.

Inclusion	Exclusion
1. Ages between 18 and 85 years	1. Patients who discontinue treatment
2. Initial APACHE II score ≥ 8 and initial EPIC score ≥ 4	2. Pregnant, lactating women, immunodeficiency, and tumor
3. First diagnosed as AP and predicted as SAP	3. AP induced by drugs and operation
4. Complete clinical and imaging data	4. Discharge without medical advice

APACHE II: Acute Physiology and Chronic Health Evaluation II; EPIC: Extra Pancreatic Inflammation on CT; AP: acute pancreatitis; SAP: severe acute pancreatitis.

**Table 2:** Univariate and multifactor analysis of Cox regression model in SAP.

	Univariate		Multifactor	
	HR (95% CI)	*p*	HR (95% CI)	*p*
Sex, man	1.101 (0.598-2.027)	0.758	─	─
Age	0.999 (0.979-1.018)	0.887	─	─
Smoke, no	0.847 (0.467-1.538)	0.586	─	─
Drink, no	0.949 (0.525-1.715)	0.862	─	─
Etiology	0.883 (0.632-1.234)	0.467	─	─
APACHE II score	1.054 (1.026-1.083)	<0.001^∗^	0.975 (0.899-1.058)	0.544
EPIC score	1.966 (1.387-2.789)	<0.001^∗^	0.959 (0.582-1.581)	0.870
Modified Marshall score	1.632 (1.411-1.887)	<0.001^∗^	1.564 (1.153-2.120)	0.004^∗^
PCT	1.006 (0.998-1.014)	0.137	─	─
CRP	1.002 (1.000-1.003)	0.010^∗^	1.001 (0.998-1.004)	0.409
WBC (∗10^9^/L)	1.052 (1.008-1.098)	0.021^∗^	1.029 (0.986-1.075)	0.188
LDH (*μ*/l)	1.001 (1.001-1.001)	<0.001^∗^	1.000 (1.000-1.001)	0.182
Ca (mmol/l)	0.238 (0.097-0.583)	0.002^∗^	1.246 (0.483-3.547)	0.680
Cr (*μ*mol/l)	1.003 (1.002-1.005)	<0.001^∗^	0.997 (0.993-1.000)	0.069
Lac	1.195 (1.052-1.358)	0.006^∗^	0.935 (0.771-1.133)	0.492
PE, no	0.026 (0.002-0.346)	0.006^∗^	0.000 (0.000-2.271*E*+95)	0.922
D-D (mg/l)	1.006 (0.974-1.040)	0.714	─	─
Albumin (g/l)	0.959 (0.900-1.021)	0.191	─	─
Amylase (*μ*/l)	1.000 (1.000-1.001)	0.020^∗^	1.000 (1.000-1.001)	0.922
APN, no	0.097 (0.023-0.400)	0.001^∗^	0.652 (0.137-3.108)	0.591
Bleeding, no	0.130 (0.072-0.233)	<0.001^∗^	0.379 (0.183-0.786)	0.009^∗^
Fungal infection, no	0.235 (0.128-0.429)	<0.001^∗^	1.042 (0.496-2.186)	0.914
ACS	0.132 (0.072-0.243)	<0.001^∗^	0.476 (0.232-0.979)	0.044^∗^
Organ failure, only one	0.059 (0.021-0.164)	<0.001^∗^	0.212 (0.065-0.696)	0.011^∗^
Thrombus, no	0.511 (0.183-1.426)	0.200	─	─

APACHE II: Acute Physiology and Chronic Health Assessment II; EPIC: Extra Pancreatic Inflammation on CT; CRP: C-reactive protein; LDH: lactate dehydrogenase; Cr: creatinine; PE: pleural effusion; ACS: abdominal compartment syndrome; ANP: acute necrosis pancreatitis. ^∗^Significant difference.

**Table 3:** Baseline characteristics of each two groups.

	Group			Group			Group		
	PE (*n* = 213)	Non-PE (*n* = 96)	*p*	IPC (*n* = 137)	Non-IPC (*n* = 76)	*p*	Group 1 (*n* = 120)	Group 2 (*n* = 17)	*p*
Sex (male)	128	64	0.31	78	50	0.21	71	7	0.16
Age (years)	51.6 ± 13.5	55.6 ± 17.0	0.03^∗^	52.4 ± 13.3	50.1 ± 13.8	0.24	52.5 ± 13.4	52 ± 13.4	0.89
Drink (%)	88 (41.3)	38 (39.6)	0.80	53 (39)	34 (44.7)	0.41	47 (39.2)	6 (39.3)	0.87
Smoke (%)	73 (34.3)	38 (39.6)	0.37	45 (33.1)	28 (36.8)	0.58	40 (33.3)	5 (29.4)	0.75
Etiology (%)			0.20			0.25			0.78
Alcohol abuse	27 (12.7)	21 (21.9)		18 (13.1)	9 (11.8)		17 (14.2)	1 (5.9)	
Gallstone	107 (50.2)	46 (47.9)		71 (51.8)	36 (47.4)		62 (51.7)	9 (52.9)	
Hyperlipemia	66 (31)	24 (25)		43 (31.4)	23 (30.3)		37 (30.8)	6 (35.3)	
Other	13 (6.1)	5 (5.2)		5 (3.7)	8 (10.5)		4 (3.3)	1 (5.9)	
APACHE II	12.7 ± 5.7	11.3 ± 3.4	0.004^∗^	11.9 ± 4.3	14.1 ± 7.4	0.02^∗^	11.7 ± 3.9	13.9 ± 6.3	0.17
EPIC score	5.1 ± 0.85	4.8 ± 0.72	<0.001^∗^	5.05 ± 0.79	5.28 ± 0.95	0.08	5.03 ± 0.79	5.18 ± 0.81	0.49
Modified Marshall score	2.99 ± 0.85	2.06 ± 0.85	<0.001^∗^	2.82 ± 1.65	3.30 ± 2.04	0.08	2.80 ± 0.1.51	2.94 ± 2.46	0.82
CRP (mg/l) Median (IQR)	226 [160-347.5]	195 [129-320]	0.06	257.6 [162.4-343]	228.1 [159-355]	0.54	242.5 [162.9-346]	242 [155.5-330]	0.86
PCT (ng/ml) Median (IQR)	2.5 [0.8-12.3]	1.83 [0.57-4.9]	0.07	2.3 [0.8-10.2]	3.4 [0.8-20.1]	0.43	2.4 [0.7-11.4]	2.2 [1.0-4.8]	0.94
LDH (*μ*/l)	937.1 ± 619.0	690.6 ± 362.3	<0.001^∗^	950.7 ± 661.0	915.4 ± 541.1	0.69	950.7 ± 661.0	915.4 ± 541.1	0.15
Cr (*μ*mol/l)	175.7 ± 156.2	105.5 ± 70.4	<0.001^∗^	157.6 ± 141.8	208.4 ± 177.1	0.02^∗^	157.6 ± 141.8	208.4 ± 177.1	0.68
Ca (mmol/l)	1.77 ± 0.31	1.93 ± 0.27	<0.001^∗^	1.79 ± 0.29	1.76 ± 0.34	0.54	1.78 ± 0.29	1.87 ± 0.23	0.23
Amylase (*μ*/l)	602.7 ± 619.6	558.3 ± 816.5	0.60	616.0 ± 598.3	578.5 ± 659.8	0.67	616.0 ± 598.3	578.5 ± 659.8	0.56
Albumin (g/l)	31.9 ± 4.6	34.2 ± 4.8	<0.001^∗^	31.9 ± 4.6	31.9 ± 4.7	0.99	31.9 ± 4.6	31.9 ± 4.7	0.39
Lac (mmol/l)	2.2 ± 1.6	1.8 ± 1.2	0.02^∗^	2.2 ± 1.7	2.3 ± 1.4	0.63	2.1 ± 1.7	2.7 ± 1.1	0.15

PE: pleural effusion; IPC: indwelling pleural catheter. Group 1: IPC within 1 week of onset. Group 2: IPC after 1 week of onset. APACHE II: Acute Physiology and Chronic Health Evaluation II; EPIC: Extra Pancreatic Inflammation on CT; CRP: C-reactive protein; PCT: procalcitonin; Cr: creatinine; IQR: interquartile range. ^∗^Significant difference.

**Table 4:** Primary and secondary endpoints of PE and non-PE groups, IPC and non-IPC groups, and group 1 and group 2.

	Group			Group			Group		
	PE (*n* = 213)	Non-PE (*n* = 96)	*p*	IPC (*n* = 137)	Non-IPC (*n* = 76)	*p*	Group 1 (*n* = 120)	Group 2 (*n* = 17)	*p*
LOS (days)	38.8 ± 25.7	20.3 ± 13.5	<0.001^∗^	38.8 ± 27.6	38.8 ± 22.0	0.99	37.1 ± 11.9	51.6 ± 21.1	0.04^∗^
Fungus infection (%)	43 (20.2)	1 (1)	<0.001^∗^	28 (20.4)	15 (19.7)	0.90	23 (19.2)	5 (29.4)	0.34
ACS (%)	60 (28.2)	9 (9.4)	<0.001^∗^	34 (24.5)	26 (34.2)	0.14	34 (24.5)	26 (34.2)	0.14
Organ failure (%)			<0.001^∗^			0.01^∗^			0.03^∗^
Single organ failure	103 (48.4)	77 (80.2)		75 (54.7)	28 (36.8)		70 (58.3)	5 (29.4)	
Multiple organ failure	110 (51.6)	19 (19.8)		62 (45.3)	48 (63.2)		50 (41.7)	12 (70.6)	
APN (%)	171 (80.3)	44 (45.8)	<0.001^∗^	104 (76.5)	66 (86.8)	0.07	88 (76.5)	16 (94.1)	0.03^∗^
Bleeding (%)	41 (19.2)	2 (2.1)	<0.001^∗^	27 (19.7)	14 (18.4)	0.82	21 (17.5)	6 (35.3)	0.10
Thrombus (%)	13 (6.1)	2 (2.1)	0.16	9 (6.6)	4 (5.3)	0.70	7 (5.8)	2 (11.8)	0.60
VST (days)	9.5 ± 13.1	2.0 ± 6.8	<0.001^∗^	9.0 ± 13.0	10.5 ± 13.4	0.43	7.6 ± 11.9	18.9 ± 16.2	0.001^∗^
PCD (%)	112 (52.5)	19 (19.8)	<0.001^∗^	65 (47.4)	47 (61.8)	0.04^∗^	50 (41.7)	15 (88.2)	<0.001^∗^
MID (%)	47 (22.1)	6 (6.3%)	0.001^∗^	32 (23.4)	15 (19.7)	0.54	23 (19.2)	9 (52.9)	0.005^∗^

PE: pleural effusion; IPC: indwelling pleural catheter. Group 1: IPC within 1 week of onset. Group 2: IPC after 1 week of onset. LOS: length of stay; ACS: abdominal compartment syndrome; ANP: acute necrosis pancreatitis; VST: ventilator supporting time; PCD: percutaneous catheter drainage; MID: minimally invasive debridement. ^∗^Significant difference.

## Data Availability

The clinical data used to support the findings of this study are restricted by the Ethics Committee of the First Affiliated Hospital of Nanchang University in order to protect patient privacy. Data are available from the Ethics Committee of the First Affiliated Hospital of Nanchang University (0791-8692505) for researchers who meet the criteria for access to confidential data.

## Supplementary Materials

Supplementary Materials The supplementary table describes the follow-up data for this study which were the patients' 60-day mortality. The 60-day mortality data of patients hospitalized for more than 60 days could be collected directly from hospitalized cases, while patients hospitalized for less than 60 days were followed up by telephone or outpatient.

## References

[1] Petrov M. S., Yadav D. (2019). Global epidemiology and holistic prevention of pancreatitis. *Nature Reviews Gastroenterology & Hepatology*.

[2] Boxhoorn L., Voermans R. P., Bouwense S. A. (2020). Acute pancreatitis. *Lancet*.

[3] Johnson C. D., Kingsnorth A. N., Imrie C. W. (2001). Double blind, randomised, placebocontrolled study of a platelet activating factor antagonist, lexipafant, in the treatment and prevention of organ failure in predicted severe acute pancreatitis. *Gut*.

[4] Heckler M., Hackert T., Hu K., Halloran C. M., Büchler M. W., Neoptolemos J. P. (2021). Severe acute pancreatitis: surgical indications and treatment. *Langenbeck's Archives of Surgery*.

[5] Kumar P., Gupta P., Rana S. (2019). Thoracic complications of pancreatitis. *JGH Open*.

[6] Shi Z., Ye W., Zhang J. (2018). LipoxinA4 attenuates acute pancreatitis-associated acute lung injury by regulating AQP-5 and MMP-9 expression, anti-apoptosis and PKC/SSeCKS-mediated F-actin activation. *Molecular Immunology*.

[7] Forsmark C. E., Swaroop Vege S., Wilcox C. M. (2016). Acute pancreatitis. *New England Journal of Medicine*.

[8] Peng R., Zhang L., Zhang Z. M., Wang Z. Q., Liu G. Y., Zhang X. M. (2020). Chest computed tomography semi-quantitative pleural effusion and pulmonary consolidation are early predictors of acute pancreatitis severity. *Quantitative Imaging in Medicine and Surgery*.

[9] Zeng Q. X., Jiang K. L., Wu Z. H. (2021). Pleural effusion is associated with severe renal dysfunction in patients with acute pancreatitis. *Medical Science Monitor*.

[10] Ng B. H., Nik Abeed N. N., Abdul Hamid M. F., Soo C. I., Low H. J., Ban A. Y. (2020). Indwelling pleural catheter and successful autopleurodesis of refractory inflammatory lupus effusion. *Respirology Case Reports*.

[11] Patil M., Dhillon S. S., Attwood K., Saoud M., Alraiyes A. H., Harris K. (2017). Management of benign pleural effusions using indwelling pleural catheters: a systematic review and meta-analysis. *Chest*.

[12] Banks P. A., Bollen T. L., Dervenis C. (2013). Classification of acute pancreatitis—2012: revision of the Atlanta classification and definitions by international consensus. *Gut*.

[13] De Waele J. J., Delrue L., Hoste E. A., De Vos M., Duyck P., Colardyn F. A. (2007). Extrapancreatic inflammation on abdominal computed tomography as an early predictor of disease severity in acute pancreatitis: evaluation of a new scoring system. *Pancreas*.

[14] Kheir F., Akulian J., Gesthalter Y. B. (2019). Indwelling tunneled pleural catheters. *American Journal of Respiratory and Critical Care Medicine*.

[15] Peery A. F., Crockett S. D., Murphy C. C. (2019). Burden and cost of gastrointestinal, liver, and pancreatic diseases in the United States: update 2018. *Gastroenterology*.

[16] Garg P. K., Singh V. P. (2019). Organ failure due to systemic injury in acute pancreatitis. *Gastroenterology*.

[17] Karki A., Riley L., Mehta H. J., Ataya A. (2019). Abdominal etiologies of pleural effusion. *Disease-a-Month*.

[18] Iyer H., Elhence A., Mittal S., Madan K., Garg P. K. (2020). Pulmonary complications of acute pancreatitis. *Expert Review of Respiratory Medicine*.

[19] Aroney N., Ure S., White H., Sane S. (2015). Recurrent undifferentiated shock: idiopathic systemic capillary leak syndrome. *Clinical Case Reports*.

[20] Suna N., Öztaş E., Kuzu U. B., Kayaçetin E., Aydoğ G., Köksal A. (2017). Pleural effusion in acute pancreatitis, not always related. *Acta Gastro-Enterologica Belgica*.

[21] Raghuwanshi S., Gupta R., Vyas M. M., Sharma R. (2016). CT evaluation of acute pancreatitis and its prognostic correlation with CT severity index. *Journal of Clinical and Diagnostic Research*.

[22] Mosztbacher D., Farkas N., Solymár M. (2017). Restoration of energy level in the early phase of acute pediatric pancreatitis. *World Journal of Gastroenterology*.

[23] Dombernowsky T., Kristensen M., Rysgaard S., Gluud L. L., Novovic S. (2016). Risk factors for and impact of respiratory failure on mortality in the early phase of acute pancreatitis. *Pancreatology*.

[24] He F., Zhu H. M., Li B. Y. (2021). Factors predicting the severity of acute pancreatitis in elderly patients. *Aging Clinical and Experimental Research*.

[25] Lee P. J., Papachristou G. I. (2019). New insights into acute pancreatitis. *Nature Reviews Gastroenterology & Hepatology*.

[26] Vege S. S., Gardner T. B., Chari S. T. (2009). Low mortality and high morbidity in severe acute pancreatitis without organ failure: a case for revising the Atlanta classification to include "moderately severe acute pancreatitis". *The American Journal of Gastroenterology*.

[27] Hines O. J., Pandol S. J. (2019). Management of severe acute pancreatitis. *BMJ*.

[28] Porcel J. M., Valencia H., Bielsa S. (2016). Factors influencing pleural drainage in parapneumonic effusions. *Revista Clínica Española*.

[29] Caronna R., Benedetti M., Morelli A. (2009). Clinical effects of laparotomy with perioperative continuous peritoneal lavage and postoperative hemofiltration in patients with severe acute pancreatitis. *World Journal of Emergency Surgery*.

[30] Xie C. L., Zhang M., Chen Y. (2018). Spleen and splenic vascular involvement in acute pancreatitis: an MRI study. *Quantitative Imaging in Medicine and Surgery*.

