# Supplementary material for: Effects of Indwelling Pleural Catheter on Severe Acute Pancreatitis: A Retrospective Study
Source: Gastroenterol Res Pract. 2022 Jan 27;2022:1919729. doi: 10.1155/2022/1919729 (PMC8813307; doi:10.1155/2022/1919729)
Supplement: Supplementary Materials — The supplementary table describes the follow-up data for this study which were the patients' 60-day mortality. The 60-day mortality data of patients hospitalized for more than 60 days could be collected directly from hospitalized cases, while patients hospitalized for less than 60 days were followed up by telephone or outpatient. [file 1919729.f1.docx]

|  | ID | Name | Sex | Age | Admission time | Tel | Follow-up way | LOS | 60days mortality |
| --- | --- | --- | --- | --- | --- | --- | --- | --- | --- |
| 1 | D01***106 | HDY | female | 77 | 2019/4/4 | 138****5498 | Clinical data and Telephone | 63 | N |
| 2 | D01***988 | WM | male | 26 | 2019/5/19 | 153****2616 | Telephone | 38 | N |
| 3 | D01***169 | LWQ | male | 42 | 2019/5/29 | / | Clinical data | 72 | Y |
| 4 | D01***247 | YYN | female | 62 | 2019/6/5 | 139****6171 | Telephone | 27 | N |
| 5 | D01***581 | ZWH | male | 44 | 2019/6/10 | 137****0899 | Telephone | 22 | N |
| 6 | D01***997 | PWP | male | 31 | 2019/6/14 | 138****1835 | Telephone | 11 | N |
| 7 | D01***643 | MSZ | male | 32 | 2019/6/17 | 188****1059 | Telephone | 20 | N |
| 8 | D01***519 | YWH | male | 52 | 2019/6/23 | / | Clinical data | 27 | Y |
| 9 | D01***117 | HH | male | 43 | 2019/7/3 | 187****6833 | Telephone | 22 | N |
| 10 | D01***807 | LYE | female | 51 | 2019/6/4 | 136****6538 | Clinical data and Telephone | 77 | N |
| 11 | D01***307 | ZW | male | 36 | 2019/7/9 | 132****3971 | Clinical data and Telephone | 65 | N |
| 12 | D01***515 | XMZ | female | 49 | 2019/8/13 | 158****7689 | Telephone | 57 | N |
| 13 | D01***922 | ZJS | male | 59 | 2019/10/19 | 159****0707 | Telephone | 38 | N |
| 14 | D01***542 | WX | male | 49 | 2019/10/24 | 135****1387 | Telephone | 23 | N |
| 15 | D01***221 | WLH | male | 57 | 2019/11/2 | / | Clinical data | 48 | Y |
| 16 | D01***291 | WXH | female | 40 | 2019/11/2 | 138****9109 | Telephone | 16 | N |
| 17 | D01***462 | HH | male | 34 | 2019/11/4 | 191****6396 | Telephone | 49 | N |
| 18 | D01***985 | XHP | male | 45 | 2019/11/11 | 151****6158 | Telephone | 40 | N |
| 19 | D01***643 | HCD | male | 81 | 2019/11/22 | 183****7553 | Telephone | 26 | N |
| 20 | D01***787 | HL | male | 30 | 2019/12/1 | / | Clinical data | 38 | Y |
| 21 | D01***299 | LP | male | 28 | 2019/12/5 | / | Clinical data | 38 | Y |
| 22 | D01***656 | XWG | male | 54 | 2019/12/6 | 189****5621 | Clinical data and Telephone | 92 | N |
| 23 | D01***773 | SD | male | 36 | 2019/12/7 | 180****0322 | Telephone | 19 | N |
| 24 | D01***122 | XPY | female | 38 | 2020/1/8 | / | Clinical data | 49 | Y |
| 25 | D01***231 | CXP | male | 54 | 2020/1/8 | 187****4330 | Clinical data and Telephone | 13 | N |
| 26 | D01***157 | LBS | male | 44 | 2020/1/12 | 185****6888 | Telephone | 11 | N |
| 27 | D01***165 | YSP | male | 43 | 2020/1/12 | 139****1949 | Telephone | 19 | N |
| 28 | D01***830 | YYZ | female | 41 | 2020/1/22 | 135****0601 | Telephone | 27 | N |
| 29 | D01***023 | FXY | female | 43 | 2020/1/23 | 182****3748 | Telephone | 32 | N |
| 30 | D01***187 | XFW | male | 31 | 2020/1/25 | / | Clinical data and Telephone | 41 | N |
| 31 | D01***112 | ZYL | male | 42 | 2020/2/7 | 135****4230 | Clinical data and Telephone | 34 | N |
| 32 | D01***632 | GML | male | 46 | 2020/2/16 | 139****4631 | Clinical data and Telephone | 78 | N |
| 33 | D01***085 | LQW | male | 40 | 2020/2/21 | / | Gastroenterology clinic | 33 | N |
| 34 | D01***851 | HAR | female | 53 | 2020/2/4 | 187****5733 | Clinical data and Telephone | 79 | N |
| 35 | D01***780 | YCX | female | 80 | 2020/3/25 | / | Gastroenterology clinic | 21 | N |
| 36 | D01***461 | LJC | male | 45 | 2020/5/17 | 137****3739 | Telephone | 11 | N |
| 37 | D09***68 | CH | female | 43 | 2018/2/22 | / | Clinical data | 118 | N |
| 38 | D09***72 | DHL | female | 50 | 2018/3/16 | / | Clinical data | 95 | N |
| 39 | D01***004 | HBG | male | 61 | 2018/5/12 | 137****9385 | Telephone | 27 | N |
| 40 | D01***347 | WXY | male | 68 | 2018/6/12 | 189****4890 | Telephone | 27 | N |
| 41 | D01***848 | JHM | female | 65 | 2018/6/29 | 138****2672 | Telephone | 10 | N |
| 42 | D01***869 | XLY | female | 68 | 2018/7/19 | / | Clinical data | 47 | Y |
| 43 | D01***610 | WXW | male | 50 | 2018/7/23 | / | Gastroenterology clinic | 16 | N |
| 44 | D01***075 | PXY | female | 42 | 2018/7/23 | / | Clinical data | 34 | Y |
| 45 | D01***719 | CLX | female | 48 | 2018/8/15 | 135****9316 | Telephone | 19 | N |
| 46 | D01***290 | ZJR | female | 56 | 2018/8/21 | 138****5073 | Telephone | 17 | N |
| 47 | D01***220 | ZXW | male | 48 | 2018/8/24 | 139****5965 | Telephone | 31 | N |
| 48 | D01***281 | HLM | female | 59 | 2018/9/7 | 182****8661 | Telephone | 33 | N |
| 49 | D01***346 | YPM | female | 84 | 2018/9/8 | 130****8901 | Telephone | 46 | N |
| 50 | D01***931 | PWP | male | 36 | 2018/9/17 | 138****4008 | Telephone | 19 | N |
| 51 | D01***368 | LHB | male | 46 | 2018/9/18 | 139****2531 | Telephone | 48 | N |
| 52 | D01***132 | XXY | female | 43 | 2018/9/28 | / | Clinical data | 94 | N |
| 53 | D01***286 | PHH | male | 31 | 2018/11/23 | / | Clinical data | 72 | N |
| 54 | D01***281 | TXH | male | 65 | 2018/12/18 | 177****2396 | Telephone | 12 | N |
| 55 | D01***868 | ZYH | male | 73 | 2019/1/26 | 188****2780 | Telephone | 15 | N |
| 56 | D01***435 | XX | male | 34 | 2019/2/2 | 130****8912 | Telephone | 24 | N |
| 57 | D01***436 | ZSL | male | 54 | 2019/2/2 | 133****5218 | Telephone | 28 | N |
| 58 | D01***358 | XJ | male | 27 | 2019/2/9 | 139****0466 | Telephone | 25 | N |
| 59 | D01***366 | HJW | male | 41 | 2019/2/9 | 152****6508 | Telephone | 19 | N |
| 60 | D01***650 | ZYW | female | 47 | 2019/2/18 | / | Clinical data | 25 | Y |
| 61 | D01***930 | HYP | female | 51 | 2019/2/19 | 159****6455 | Telephone | 48 | N |
| 62 | D01***487 | LSY | female | 61 | 2019/2/20 | / | Clinical data | 70 | N |
| 63 | D01***355 | JYP | male | 64 | 2019/2/23 | 139****2055 | Telephone | 37 | N |
| 64 | D01***484 | WSZ | female | 67 | 2019/2/26 | / | Clinical data | 7 | Y |
| 65 | D01***516 | SRF | male | 46 | 2019/3/1 | / | Clinical data | 11 | Y |
| 66 | D01***210 | HZB | male | 37 | 2019/4/5 | 150****1665 | Telephone | 18 | N |
| 67 | D01***465 | GSY | male | 46 | 2019/4/12 | / | Gastroenterology clinic | 49 | N |
| 68 | D01***079 | QYC | male | 46 | 2019/4/15 | / | Clinical data | 23 | N |
| 69 | D01***400 | PHE | male | 45 | 2019/4/15 | 157****2302 | Telephone | 29 | N |
| 70 | D01***308 | HT | male | 32 | 2019/4/17 | / | Clinical data | 62 | N |
| 71 | D01***162 | ZXL | female | 56 | 2019/4/26 | / | Clinical data | 84 | N |
| 72 | D01***679 | ZZM | female | 59 | 2019/5/2 | 136****1732 | Telephone | 20 | N |
| 73 | D09***27 | ZMJ | male | 76 | 2017/8/10 | 138****9335 | Telephone | 24 | N |
| 74 | D09***04 | LFG | male | 61 | 2017/12/19 | / | Clinical data | 22 | N |
| 75 | D09***28 | WDL | female | 47 | 2017/12/26 | / | Clinical data | 138 | N |
| 76 | D09***24 | LXL | female | 51 | 2017/12/27 | 131****5698 | Telephone | 45 | N |
| 77 | D09***17 | YJE | female | 57 | 2017/12/30 | / | Clinical data | 70 | N |
| 78 | D09***86 | YSW | female | 68 | 2018/1/2 | 150****5070 | Telephone | 15 | N |
| 79 | D09***58 | LYY | female | 71 | 2018/1/2 | / | Gastroenterology clinic | 37 | N |
| 80 | D09***09 | SL | male | 35 | 2018/1/9 | / | Clinical data | 56 | Y |
| 81 | D09***77 | ZZQ | male | 37 | 2018/2/20 | 187****7208 | Telephone | 14 | N |
| 82 | D01***324 | CSY | female | 50 | 2020/5/8 | / | Clinical data | 60 | N |
| 83 | D01***917 | XR | male | 35 | 2020/6/7 | / | Clinical data | 99 | N |
| 84 | D01***157 | WHK | male | 75 | 2020/6/23 | 180****9633 | Telephone | 23 | N |
| 85 | D01***790 | FGZ | male | 38 | 2020/6/27 | 182****5392 | Telephone | 12 | N |
| 86 | D01***437 | HQS | male | 34 | 2020/6/29 | 159****1910 | Gastroenterology clinic and Telephone | 11 | N |
| 87 | D01***238 | WRZ | male | 43 | 2020/7/9 | / | Clinical data | 14 | Y |
| 88 | D01***067 | WMN | female | 69 | 2020/7/16 | / | Clinical data | 20 | N |
| 89 | D01***351 | CLL | female | 43 | 2020/8/1 | 151****3271 | Telephone | 30 | N |
| 90 | D01***366 | HFY | male | 43 | 2020/8/2 | / | Clinical data | 32 | Y |
| 91 | D12***40 | WLD | female | 67 | 2020/8/17 | 153****1918 | Telephone | 23 | N |
| 92 | D01***479 | ZJY | male | 81 | 2020/8/20 | 139****0266 | Telephone | 11 | N |
| 93 | D01***589 | FHX | female | 76 | 2020/9/30 | 137****1760 | Telephone | 36 | N |
| 94 | D01***863 | XC | male | 30 | 2020/10/3 | 188****3777 | Telephone | 20 | N |
| 95 | D01***387 | DCS | male | 68 | 2020/10/8 | / | Clinical data | 24 | N |
| 96 | D01***821 | XSX | male | 62 | 2020/10/28 | / | Clinical data | 27 | N |
| 97 | D09***57 | WNJ | female | 67 | 2019/5/9 | / | Clinical data | 26 | N |
| 98 | D01***748 | WAL | female | 46 | 2019/6/17 | 134****9588 | Telephone | 55 | N |
| 99 | D01***206 | LAX | male | 18 | 2020/3/16 | / | Gastroenterology clinic | 36 | N |
| 100 | D01***874 | ZYZ | male | 80 | 2018/8/20 | 137****0755 | Telephone | 25 | N |
| 101 | D01***711 | WRD | male | 53 | 2018/12/20 | / | Clinical data | 77 | N |
| 102 | D01***394 | XZS | male | 63 | 2019/3/29 | 183****5645 | Telephone | 12 | N |
| 103 | D01***558 | XLG | male | 47 | 2019/5/20 | 134****2888 | Telephone | 22 | N |
| 104 | D06***69 | XLH | male | 41 | 2019/3/26 | / | Clinical data | 76 | N |
| 105 | D09***74 | QCS | male | 51 | 2017/12/19 | / | Clinical data | 101 | N |
| 106 | D09***46 | FAJ | female | 56 | 2018/1/8 | / | Clinical data | 11 | Y |
| 107 | D09***09 | PSF | male | 43 | 2018/1/15 | 186****2839 | Telephone | 11 | N |
| 108 | D09***05 | XXM | female | 67 | 2018/1/27 | 152****4508 | Telephone | 20 | N |
| 109 | D09***50 | XYL | female | 71 | 2018/2/15 | / | Gastroenterology clinic | 20 | N |
| 110 | D09***56 | CJG | male | 53 | 2018/2/28 | 136****0440 | Telephone | 14 | N |
| 111 | D09***05 | SH | male | 48 | 2018/2/27 | / | Clinical data | 25 | Y |
| 112 | D09***39 | JLR | male | 36 | 2018/2/28 | 137****2520 | Telephone | 26 | N |
| 113 | D09***94 | JLM | female | 65 | 2018/4/2 | 138****1204 | Telephone | 22 | N |
| 114 | D09***29 | XJG | male | 74 | 2021/3/31 | 151****0918 | Telephone | 24 | N |
| 115 | D09***01 | FLY | male | 72 | 2018/4/24 | / | Clinical data | 29 | Y |
| 116 | D09***56 | XXB | male | 43 | 2018/4/27 | / | Gastroenterology clinic | 46 | N |
| 117 | D01***211 | LCH | female | 69 | 2018/5/25 | 130****1179 | Telephone | 20 | N |
| 118 | D01***607 | DCS | male | 58 | 2018/6/25 | / | Clinical data | 20 | Y |
| 119 | D01***161 | ZWC | male | 50 | 2018/6/23 | 150****7382 | Telephone | 27 | N |
| 120 | D01***709 | YYX | male | 69 | 2018/7/23 | 158****0535 | Telephone | 12 | N |
| 121 | D01***569 | WDL | female | 68 | 2018/7/24 | 152****3156 | Telephone | 12 | N |
| 122 | D01***108 | CR | female | 47 | 2018/8/9 | / | Clinical data | 8 | Y |
| 123 | D01***457 | LXH | female | 51 | 2018/8/15 | 151****8600 | Telephone | 23 | N |
| 124 | D01***498 | CWH | male | 45 | 2018/8/26 | 153****2717 | Telephone | 19 | N |
| 125 | D01***064 | HQZ | male | 68 | 2018/9/10 | 186****3399 | Telephone | 17 | N |
| 126 | D01***398 | TMX | female | 74 | 2018/9/30 | / | Clinical data | 14 | N |
| 127 | D01***239 | HYM | male | 45 | 2018/10/10 | / | Clinical data | 12 | N |
| 128 | D01***799 | RCJ | male | 28 | 2018/10/18 | / | Clinical data | 63 | N |
| 129 | DO1***893 | LFY | female | 61 | 2018/12/17 | 136****8683 | Telephone | 59 | N |
| 130 | D01***402 | LGH | female | 40 | 2018/11/3 | / | Clinical data | 87 | N |
| 131 | D01***162 | GYH | female | 33 | 2018/12/18 | 173****4887 | Telephone | 10 | N |
| 132 | D01***900 | XDX | female | 65 | 2018/11/27 | 130****2366 | Telephone | 51 | N |
| 133 | DO1***543 | JZT | male | 25 | 2018/12/5 | 137****1359 | Telephone | 14 | N |
| 134 | D01***692 | HGH | male | 67 | 2018/12/2 | 188****3308 | Telephone | 33 | N |
| 135 | D01***958 | ZJH | male | 67 | 2018/11/1 | 180****9507 | Telephone | 32 | N |
| 136 | D01***065 | DCL | male | 42 | 2018/10/22 | 138****9688 | Telephone | 50 | N |
| 137 | D01***893 | LFY | female | 61 | 2018/12/17 | / | Clinical data | 60 | N |
| 138 | D01***642 | LYQ | female | 61 | 2019/1/5 | 159****1963 | Telephone | 10 | N |
| 139 | D01***168 | XXH | male | 32 | 2019/1/2 | / | Clinical data | 109 | N |
| 140 | D01***172 | LZP | male | 44 | 2019/1/6 | 159****0138 | Telephone | 56 | N |
| 141 | D01***898 | ZMF | male | 55 | 2019/2/6 | / | Clinical data | 100 | N |
| 142 | D01***913 | DWL | male | 41 | 2019/3/17 | 139****6182 | Telephone | 16 | N |
| 143 | D01***886 | YAM | female | 63 | 2019/2/11 | / | Clinical data | 18 | Y |
| 144 | D01***991 | LLS | male | 31 | 2019/2/7 | / | Clinical data | 34 | Y |
| 145 | D01***873 | ZF | female | 34 | 2019/2/12 | / | Clinical data | 105 | N |
| 146 | D01***477 | WPA | male | 58 | 2019/2/26 | / | Clinical data | 13 | Y |
| 147 | D01***002 | WXZ | female | 54 | 2019/3/22 | / | Clinical data | 8 | Y |
| 148 | D01***233 | LC | male | 23 | 2019/3/17 | 139****6182 | Telephone | 23 | N |
| 149 | D01***727 | HSY | female | 65 | 2019/4/8 | 158****9787 | Telephone | 10 | N |
| 150 | D01***071 | YAQ | female | 64 | 2019/2/19 | / | Clinical data | 63 | N |
| 151 | D01***536 | XPX | male | 74 | 2019/4/9 | / | Clinical data | 23 | Y |
| 152 | D01***163 | HJX | female | 39 | 2019/3/22 | / | Clinical data | 62 | N |
| 153 | D01***393 | MT | male | 26 | 2019/5/10 | 130****7393 | Telephone | 13 | N |
| 154 | D01***429 | WDS | male | 62 | 2019/4/15 | 137****3992 | Telephone | 52 | N |
| 155 | D01***585 | GJY | female | 77 | 2019/5/20 | / | Clinical data | 16 | N |
| 156 | D01***369 | LZG | male | 48 | 2019/5/29 | 189****5581 | Telephone | 20 | N |
| 157 | D01***389 | CJS | male | 64 | 2019/6/28 | / | Clinical data | 8 | N |
| 158 | D01***967 | LYT | male | 51 | 2019/6/29 | 182****3509 | Telephone | 10 | N |
| 159 | D01***724 | HLW | male | 67 | 2019/6/24 | 135****1331 | Telephone | 24 | N |
| 160 | D01***331 | WH | male | 61 | 2019/7/4 | 139****4554 | Telephone | 25 | N |
| 161 | D01***996 | HFW | male | 46 | 2019/7/21 | 176****8892 | Telephone | 8 | N |
| 162 | D01***583 | LX | male | 33 | 2019/7/22 | 139****9687 | Telephone | 10 | N |
| 163 | D01***585 | LMN | female | 68 | 2019/7/31 | / | Clinical data | 7 | Y |
| 164 | D01***089 | ZHS | male | 59 | 2019/7/26 | / | Clinical data | 15 | Y |
| 165 | D01***735 | GY | male | 53 | 2019/7/19 | / | Clinical data | 31 | Y |
| 166 | D01***446 | ZGP | male | 38 | 2019/6/25 | / | Clinical data | 63 | N |
| 167 | D01***681 | GXY | female | 57 | 2019/8/7 | 139****6968 | Telephone | 27 | N |
| 168 | D01***268 | LZH | female | 56 | 2019/8/7 | 154****8174 | Telephone | 26 | N |
| 169 | D01***236 | WT | male | 25 | 2019/10/27 | 182****0696 | Telephone | 9 | N |
| 170 | D01***979 | YCX | female | 30 | 2019/9/6 | 135****2574 | Telephone | 26 | N |
| 171 | D01***940 | GZQ | male | 38 | 2019/8/14 | / | Clinical data | 63 | N |
| 172 | D01***869 | ZXX | female | 67 | 2019/9/6 | 159****6030 | Telephone | 40 | N |
| 173 | D01***203 | XHX | female | 70 | 2019/9/24 | 137****4670 | Telephone | 22 | N |
| 174 | D01***942 | YRG | male | 62 | 2019/9/23 | / | Clinical data | 26 | Y |
| 175 | D01***439 | JXH | male | 84 | 2019/8/19 | / | Clinical data | 71 | N |
| 176 | D01***138 | LJF | female | 66 | 2019/9/10 | / | Telephone | 51 | N |
| 177 | D01***809 | WCH | female | 65 | 2019/10/18 | 136****9204 | Telephone | 17 | N |
| 178 | D01***219 | LX | male | 34 | 2019/10/29 | 136****7589 | Telephone | 26 | N |
| 179 | D01***904 | WMT | male | 53 | 2019/9/19 | 189****1980 | Telephone | 57 | N |
| 180 | D01***598 | LQH | female | 58 | 2019/9/22 | / | Clinical data | 58 | Y |
| 181 | D01***006 | WQM | male | 53 | 2019/11/7 | 182****7750 | Telephone | 12 | N |
| 182 | D01***829 | FDH | female | 76 | 2019/11/11 | / | Clinical data | 15 | Y |
| 183 | D01***829 | MXS | male | 64 | 2019/11/13 | 152****7259 | Telephone | 19 | N |
| 184 | D01***008 | WLX | male | 53 | 2019/11/18 | 182****0968 | Telephone | 10 | N |
| 185 | D01***503 | XXF | female | 68 | 2019/11/27 | / | Clinical data | 27 | Y |
| 186 | D01***857 | XCF | female | 51 | 2019/12/21 | 159****7057 | Telephone | 27 | N |
| 187 | D01***559 | SAF | female | 76 | 2019/12/19 | 150****3743 | Telephone | 35 | N |
| 188 | D01***787 | NLQ | female | 69 | 2019/12/7 | 185****7925 | Telephone | 53 | N |
| 189 | D01***793 | CLH | male | 49 | 2020/2/26 | / | Clinical data | 63 | N |
| 190 | D01***851 | LYS | male | 43 | 2020/4/7 | 138****6326 | Telephone and Gastroenterology clinic | 16 | N |
| 191 | D01***941 | WJH | male | 37 | 2020/4/5 | 151****0139 | Telephone and Gastroenterology clinic | 20 | N |
| 192 | D01***145 | HDL | male | 56 | 2020/3/26 | / | Gastroenterology clinic | 42 | N |
| 193 | D01***819 | LWD | male | 46 | 2020/4/4 | 157****5812 | Telephone | 35 | N |
| 194 | D01***935 | WF | male | 35 | 2020/5/7 | 188****6829 | Telephone | 18 | N |
| 195 | D01***436 | ZJR | female | 54 | 2020/5/1 | 136****0897 | Telephone | 28 | N |
| 196 | D01***352 | WGJ | female | 69 | 2020/5/9 | 133****0890 | Telephone | 20 | N |
| 197 | D01***675 | ZDY | female | 47 | 2020/5/21 | 135****3634 | Telephone | 14 | N |
| 198 | D01***489 | ZMR | female | 72 | 2020/5/17 | 134****9269 | Telephone | 26 | N |
| 199 | D01***263 | KJS | male | 57 | 2020/6/11 | / | Clinical data | 12 | Y |
| 200 | D01***350 | ZYF | female | 51 | 2020/4/6 | / | Clinical data | 73 | N |
| 201 | D01***525 | TMG | female | 67 | 2020/6/5 | 135****9431 | Telephone | 17 | N |
| 202 | D01***482 | ZHN | female | 71 | 2020/6/12 | 158****1925 | Telephone | 12 | N |
| 203 | D01***792 | FLF | male | 84 | 2020/6/14 | 138****2182 | Telephone | 10 | N |
| 204 | D01***695 | HYZ | female | 55 | 2020/6/9 | 133****5670 | Telephone | 23 | N |
| 205 | D01***790 | FGZ | male | 38 | 2020/6/27 | 187****2021 | Telephone | 12 | N |
| 206 | D01***095 | YZY | female | 18 | 2020/5/22 | 152****6718 | Telephone | 52 | N |
| 207 | D01***369 | WLH | female | 42 | 2020/7/17 | 138****7525 | Telephone | 20 | N |
| 208 | D01***550 | LLY | female | 72 | 2020/7/26 | 139****8606 | Telephone | 11 | N |
| 209 | D01***489 | GKW | male | 54 | 2020/7/26 | / | Clinical data | 11 | Y |
| 210 | D01***113 | WJQ | male | 85 | 2020/7/20 | 139****9823 | Telephone | 30 | N |
| 211 | D01***023 | QHQ | male | 30 | 2020/5/15 | / | Clinical data | 98 | N |
| 212 | D01***014 | HSY | male | 43 | 2020/6/15 | / | Clinical data | 72 | N |
| 213 | D01***288 | cwz | male | 63 | 2020/8/12 | / | Clinical data | 16 | Y |
| 214 | D01***540 | WLD | female | 66 | 2020/8/17 | 153****1918 | Telephone | 23 | N |
| 215 | D01***637 | WYH | female | 72 | 2020/9/17 | 183****0356 | Telephone | 21 | N |
| 216 | D01***055 | LYX | female | 73 | 2020/8/12 | / | Clinical data | 62 | N |
| 217 | D01***200 | XXZ | female | 56 | 2020/9/12 | / | Clinical data | 32 | Y |
| 218 | D01***475 | FWX | male | 50 | 2020/9/21 | 158****4353 | Telephone | 54 | N |
| 219 | D01***177 | LA | male | 34 | 2020/10/11 | / | Clinical data | 33 | Y |
| 220 | D01***681 | ZH | female | 72 | 2020/10/9 | / | Gastroenterology clinic | 42 | N |
| 221 | D01***037 | YXJ | male | 45 | 2020/10/30 | 137****1815 | Telephone | 14 | N |
| 222 | D01***479 | RDH | male | 44 | 2020/9/6 | / | Clinical data | 85 | N |
| 223 | D01***917 | FJF | male | 59 | 2020/11/6 | / | Gastroenterology clinic | 31 | N |
| 224 | D01***598 | THC | male | 75 | 2020/11/13 | 139****5297 | Telephone | 25 | N |
| 225 | D01***986 | ZJY | female | 68 | 2020/10/22 | / | Gastroenterology clinic | 40 | N |
| 226 | D01***169 | HLL | male | 63 | 2020/12/1 | / | Gastroenterology clinic | 14 | N |
| 227 | D01***547 | LCY | female | 65 | 2020/12/2 | 151****6988 | Telephone | 16 | N |
| 228 | D01***704 | HCC | male | 82 | 2020/12/16 | / | Gastroenterology clinic | 7 | N |
| 229 | D01***980 | HMQ | male | 37 | 2020/11/2 | / | Clinical data | 52 | N |
| 230 | D01***972 | SWY | female | 53 | 2020/11/30 | / | Clinical data | 72 | N |
| 231 | D01***784 | CYY | female | 79 | 2020/12/16 | 177****5022 | Telephone | 12 | N |
| 232 | D01***547 | MSQ | male | 40 | 2020/11/29 | / | Clinical data | 50 | N |
| 233 | D01***434 | WF | male | 35 | 2020/12/19 | / | Clinical data | 79 | N |
| 234 | D01***453 | QHW | male | 54 | 2020/12/19 | / | Clinical data | 24 | Y |
| 235 | D01***530 | RAL | female | 55 | 2020/12/20 | / | Gastroenterology clinic | 11 | N |
| 236 | D08***99 | HJR | male | 70 | 2016/12/21 | 152****1555 | Telephone | 12 | N |
| 237 | D08***76 | ZWG | male | 42 | 2016/12/24 | 138****1021 | Telephone | 19 | N |
| 238 | D08***44 | XRG | male | 72 | 2016/12/24 | 138****1062 | Telephone | 20 | N |
| 239 | D08***51 | ZH | male | 47 | 2016/10/26 | 156****6600 | Telephone | 33 | N |
| 240 | D08***90 | WXJ | male | 47 | 2016/11/26 | / | Clinical data | 36 | Y |
| 241 | D08***78 | NJH | male | 48 | 2017/1/1 | / | Clinical data | 11 | Y |
| 242 | D08***47 | ZSY | female | 54 | 2017/1/13 | 186****9681 | Telephone | 14 | N |
| 243 | D08***71 | XFM | female | 78 | 2017/1/17 | 180****4829 | Telephone | 16 | N |
| 244 | D08***36 | XAM | female | 34 | 2017/1/1 | / | Clinical data | 56 | Y |
| 245 | D08***03 | SHH | male | 58 | 2017/1/14 | 181****8889 | Telephone | 37 | N |
| 246 | D08***90 | GXL | male | 49 | 2017/5/16 | 159****7346 | Telephone | 17 | N |
| 247 | DO8***66 | QXX | male | 49 | 2017/2/22 | 159****3160 | Telephone | 9 | N |
| 248 | D08***28 | XJ | male | 32 | 2017/2/5 | 159****1888 | Telephone | 27 | N |
| 249 | D08***69 | WLT | male | 83 | 2017/3/14 | 139****6247 | Telephone | 8 | N |
| 250 | D08***29 | WLX | female | 43 | 2017/2/2 | 152****5186 | Telephone | 51 | N |
| 251 | D08***19 | PXX | male | 52 | 2017/3/24 | 158****0156 | Telephone | 14 | N |
| 252 | D08***55 | XQX | female | 65 | 2017/3/4 | 137****0358 | Telephone | 45 | N |
| 253 | D08***88 | ZWW | female | 28 | 2017/3/19 | 132****7518 | Telephone | 33 | N |
| 254 | D08***29 | XGP | male | 70 | 2017/3/21 | / | Clinical data | 24 | Y |
| 255 | D08***97 | ZSS | male | 69 | 2017/4/9 | 139****5794 | Telephone | 11 | N |
| 256 | D08***90 | WZY | female | 73 | 2017/4/9 | 138****5326 | Telephone | 16 | N |
| 257 | D08***91 | ZB | male | 48 | 2017/4/13 | 138****6655 | Telephone | 19 | N |
| 258 | D08***11 | ZDM | female | 57 | 2017/4/5 | 182****3973 | Telephone | 29 | N |
| 259 | D08***08 | LDL | female | 75 | 2017/4/21 | 139****7351 | Telephone | 18 | N |
| 260 | D08***05 | ZQH | male | 25 | 2017/2/14 | / | Clinical data | 85 | N |
| 261 | D08***03 | CHM | male | 65 | 2017/5/1 | 139****3788 | Telephone | 9 | N |
| 262 | D08***90 | PFJ | male | 56 | 2017/4/17 | 186****0109 | Telephone | 29 | N |
| 263 | D08***24 | ZML | male | 53 | 2017/5/6 | 150****9592 | Telephone | 27 | N |
| 264 | D08***64 | LHG | male | 51 | 2017/5/16 | 151****3090 | Telephone | 18 | N |
| 265 | D08***66 | ZSF | male | 53 | 2017/4/26 | 132****7271 | Telephone | 38 | N |
| 266 | D08***14 | XDG | male | 54 | 2017/5/15 | 151****7834 | Telephone | 23 | N |
| 267 | D09***03 | CHX | female | 32 | 2017/5/24 | 188****6136 | Telephone | 16 | N |
| 268 | D09***99 | ZYH | female | 63 | 2017/5/29 | 139****2170 | Telephone | 14 | N |
| 269 | D08***56 | WBM | male | 53 | 2017/4/7 | / | Clinical data | 82 | N |
| 270 | D08***66 | ZZM | female | 62 | 2017/4/15 | / | Clinical data | 62 | N |
| 271 | D09***04 | HWM | female | 74 | 2017/6/2 | 182****9396 | Telephone | 18 | N |
| 272 | D09***41 | LWT | male | 45 | 2017/6/3 | 187****5578 | Telephone | 17 | N |
| 273 | D09***73 | ZXW | male | 42 | 2017/5/25 | 156****3200 | Telephone | 40 | N |
| 274 | D09***45 | CYB | male | 56 | 2017/5/29 | 133****1096 | Telephone | 43 | N |
| 275 | D09***35 | ZQH | male | 45 | 2017/6/11 | 135****2949 | Telephone | 55 | N |
| 276 | D09***57 | YDZ | male | 69 | 2017/7/9 | 187****5118 | Telephone | 20 | N |
| 277 | D09***79 | ZWW | male | 26 | 2017/7/10 | 158****2835 | Telephone | 23 | N |
| 278 | D09***97 | LSC | male | 49 | 2017/7/24 | 136****3466 | Telephone | 11 | N |
| 279 | D09***85 | FDF | male | 77 | 2017/7/31 | 135****4412 | Telephone | 7 | N |
| 280 | D09***59 | JLL | male | 50 | 2017/7/25 | 189****3508 | Telephone | 16 | N |
| 281 | D09***54 | WF | male | 39 | 2017/7/5 | 152****8999 | Telephone | 40 | N |
| 282 | D09***92 | HSL | male | 65 | 2017/7/26 | 150****9511 | Telephone | 20 | N |
| 283 | D09***87 | YDX | female | 44 | 2017/7/31 | 151****2861 | Telephone | 16 | N |
| 284 | D09***14 | GXM | female | 68 | 2017/8/4 | 135****2772 | Telephone | 21 | N |
| 285 | D09***25 | LYL | male | 73 | 2017/8/3 | 158****6850 | Telephone | 18 | N |
| 286 | D09***51 | DMJ | male | 55 | 2017/8/7 | 138****4845 | Telephone | 14 | N |
| 287 | D09***90 | CQX | male | 50 | 2017/8/8 | 139****7839 | Telephone | 14 | N |
| 288 | D09***69 | YXR | male | 62 | 2017/8/21 | 189****7406 | Telephone | 11 | N |
| 289 | D09***72 | YHX | female | 65 | 2017/7/28 | / | Clinical data | 74 | N |
| 290 | D09***51 | LJL | female | 65 | 2017/8/29 | 138****5119 | Telephone | 13 | N |
| 291 | D09***75 | AXS | male | 54 | 2017/8/24 | 187****9966 | Telephone | 27 | N |
| 292 | D09***92 | WL | male | 32 | 2017/9/4 | 159****9093 | Telephone | 15 | N |
| 293 | D09***47 | ZCD | male | 51 | 2017/7/29 | 159****0198 | Telephone | 56 | N |
| 294 | D09***43 | WMP | female | 25 | 2017/9/25 | 131****1682 | Telephone | 12 | N |
| 295 | D09***55 | LWL | female | 41 | 2017/9/6 | 137****8661 | Telephone | 33 | N |
| 296 | D09***65 | LHY | female | 45 | 2017/9/13 | 158****5062 | Telephone | 26 | N |
| 297 | D09***34 | PZS | male | 78 | 2017/9/21 | 134****3747 | Telephone | 19 | N |
| 298 | D09***30 | DSL | female | 59 | 2017/10/6 | 138****0393 | Telephone | 22 | N |
| 299 | D09***09 | LWL | male | 29 | 2017/10/16 | 182****8273 | Telephone | 15 | N |
| 300 | D09***72 | HZH | male | 40 | 2017/10/17 | / | Clinical data | 14 | Y |
| 301 | D09***62 | HSS | male | 46 | 2017/9/12 | / | Clinical data | 44 | Y |
| 302 | D09***49 | LHD | male | 56 | 2017/9/27 | 135****2129 | Telephone | 48 | N |
| 303 | D09***94 | HFG | male | 74 | 2017/11/9 | 139****4006 | Telephone | 18 | N |
| 304 | D09***55 | TLY | female | 68 | 2017/11/2 | / | Clinical data | 14 | Y |
| 305 | D09***24 | LY | male | 21 | 2017/11/28 | 180****3333 | Telephone | 10 | N |
| 306 | D09***62 | DCL | female | 55 | 2017/11/5 | / | Clinical data | 69 | N |
| 307 | D09***75 | CRP | male | 41 | 2017/12/6 | / | Clinical data | 31 | Y |
| 308 | D09***09 | CYH | male | 49 | 2017/12/7 | 131****3475 | Telephone | 18 | N |
| 309 | D09***05 | SHH | male | 52 | 2017/12/1 | / | Clinical data | 125 | N |
